# Supplementary material for: The genome sequence of the Olive Crescent, Trisateles emortualis (Denis & Schiffermüller, 1775)
Source: Wellcome Open Res. 2024 Apr 8;9:178. [Version 1] doi: 10.12688/wellcomeopenres.20836.1 (PMC11862366; doi:10.12688/wellcomeopenres.20836.1)
Supplement: Peer review history

# Reviewer report: Reviewer response for version 1

**DOI:** 10.21956/wellcomeopenres.23055.r105735

**Published:** 2025-02-25

**Authors:** Kay Lucek

**Affiliations:** 1 University of Neuchâtel, Neuchâtel, Switzerland

The authors present the chromosome level genome assembly of a male specimen of the Olive Crescent moth ( *Trisateles emortualis*). The assembly consists of 31 chromosomes including the Z chromosome. The assembly is highly complete as revealed by the high BUSCO score but not fully phased. Sequencing and genome assembly follow the current state of the art and use established methods.

 Overall, the presented assembly will be of great value to resolve the taxonomic status of this species, genus and family. Moreover, *Trisateles emortualis *feeds on dead and withered leaves, which is not very common. Therefore this reference genome could provide unique insights into this special adaptation.

Are sufficient details of methods and materials provided to allow replication by others?

Yes

Is the rationale for creating the dataset(s) clearly described?

Yes

Are the datasets clearly presented in a useable and accessible format?

Yes

Are the protocols appropriate and is the work technically sound?

Yes

Reviewer Expertise:

Biodiversity Genomics

I confirm that I have read this submission and believe that I have an appropriate level of expertise to confirm that it is of an acceptable scientific standard.

# Reviewer report: Reviewer response for version 1

**DOI:** 10.21956/wellcomeopenres.23055.r91732

**Published:** 2024-08-12

**Authors:** Steven Van Belleghem

**Affiliations:** 1 Ecology, Evolution and Conservation Biology, Biology Department, University of Leuven, Rio Piedras, Leuven, Belgium

In this report, the genome sequence of the Olive Crescent is presented. The species and its characteristics are well introduced. Using PacBio and Hi-C this genome is assembled into 31 chromosomal pseudomolecules with 13,176 identified protein coding genes. The genome assembly statistics indicate the assembly is of high quality, similar to other genomes presented by Tree of Life consortium.

Are sufficient details of methods and materials provided to allow replication by others?

Yes

Is the rationale for creating the dataset(s) clearly described?

Yes

Are the datasets clearly presented in a useable and accessible format?

Yes

Are the protocols appropriate and is the work technically sound?

Yes

Reviewer Expertise:

Population genomics

I confirm that I have read this submission and believe that I have an appropriate level of expertise to confirm that it is of an acceptable scientific standard.
